# Dietary fish intake increased the concentration of soluble ACE2 in rats: can fish consumption reduce the risk of COVID-19 infection through interception of SARS-CoV-2 by soluble ACE2?

**DOI:** 10.1017/S0007114523000776

**Published:** 2023-11-28

**Authors:** Maria O’Keeffe, Åge Oterhals, Linn Anja Slåke Vikøren, Aslaug Drotningsvik, Gunnar Mellgren, Alfred Halstensen, Oddrun Anita Gudbrandsen

**Affiliations:** 1 Dietary Protein Research Group, Centre for Nutrition, Department of Clinical Medicine, University of Bergen, Bergen 5021, Norway; 2 Nofima, P.B. 1425 Oasen, Bergen 5844, Norway; 3 Mohn Nutrition Research Laboratory, Department of Clinical Science, University of Bergen, Bergen 5021, Norway; 4 Hormone Laboratory, Department of Medical Biochemistry and Pharmacology, Haukeland University Hospital, Bergen 5021, Norway; 5 Department of Clinical Science, University of Bergen, 5021 Bergen, Norway; 6 K. Halstensen AS, P.O. Box 103, Bekkjarvik 5399, Norway

**Keywords:** COVID-19, Angiotensin-converting enzyme-2, Atlantic cod, Soluble ACE2

## Abstract

The severe acute respiratory syndrome coronavirus 2 (SARS-CoV-2) enters the cells after binding to the membrane-bound receptor angiotensin-converting enzyme 2 (ACE2), but this may be prevented through interception by soluble ACE2 (sACE2) or by inhibition of the ACE2 receptor, thus obstructing cell entry and replication. The main objective of this study was to investigate if fish intake affected the concentration of sACE2 in rats. The secondary aim was to evaluate the *in vitro* ACE2-inhibiting activity of fish proteins. Rats were fed cod muscle as 25 % of dietary protein, and blood was collected after 4 weeks of intervention. Muscle, backbone, skin, head, stomach, stomach content, intestine and swim bladder from haddock, saithe, cod and redfish were hydrolysed with trypsin before ACE2-inhibiting activity was measured *in vitro*. *In vivo* data were compared using unpaired Student’s *t* test, and *in vitro* data were compared using one-way ANOVA followed by the Tukey HSD *post hoc* test. The mean sACE2 concentration was 47 % higher in rats fed cod when compared with control rats (P 0·034), whereas serum concentrations of angiotensin II and TNF-*α* were similar between the two experimental groups. Muscle, backbone, skin and head from all four fish species inhibited ACE2 activity *in vitro*, whereas the remaining fractions had no effect. To conclude, our novel data demonstrate that fish intake increased the sACE2 concentration in rats and that the hydrolysed fish proteins inhibited ACE2 activity *in vitro*.

The spike protein of the severe acute respiratory syndrome coronavirus 2 (SARS-CoV-2) binds to angiotensin-converting enzyme 2 (ACE2) on the cell surface^(1)^ and is activated by transmembrane protease serine 2 (TMPRSS2)^(2)^. The entry of SARS-CoV-2 into cells may be blocked by inhibition of TMPRSS2^(1)^, but as yet, the effect of inhibiting ACE2 for the prevention of SARS-CoV-2 infection is not known. The major route of entry for SARS-CoV-2 is through the respiratory tract, and coexpression of ACE2 and TMPRSS2 has been found in cells in the nasal and buccal mucosa and in the epithelia of bronchus and larynx^(3)^. The risk for a severe course including death from coronavirus disease 2019 (COVID-19) after infection with SARS-CoV-2 is increased in persons with obesity^(4,5)^ and is in line with the observation of elevated expressions of ACE2 and TMPRSS2 in the trachea of obese mice^(6)^.

In addition to its function as a receptor, ACE2 is also involved in several processes, such as regulation of blood pressure, fibrosis, inflammation and thrombosis^(7)^. ACE2 is a relatively newly identified human metalloprotease that shares a homology in amino acid sequence of about 40 % with angiotensin-converting enzyme (ACE)^(8,9)^. Both ACE2 and ACE are important players in the renin–angiotensin system, which is a central regulator of blood pressure as well as the fluid and electrolyte balance. ACE2 catalyses the removal of the carboxyterminal amino acid from angiotensin II to produce the vasodilator angiotensin-(1–7), and, with less affinity, removes the carboxyterminal leucyl residue from angiotensin I to produce angiotensin (1–9)^(7)^. ACE, on the other hand, removes the C-terminal dipeptide (histidyl-leucine) from angiotensin I to produce the vasoconstrictor angiotensin II and also removes the terminal dipeptide (phenylalanyl-histidine) from angiotensin (1–9) to produce angiotensin (1–7) and hydrolyses the vasodilator bradykinin^(7)^. Thus, ACE2 partly counteracts the effects of ACE by contributing to both the production of the vasodilator angiotensin 1–7 and decreases the availability of angiotensin I as a precursor for the vasoconstrictor angiotensin II through ACE activity, and consequently, upregulation of ACE2 activity may counteract some of the unfavourable effects of ACE. ACE2 is believed to have a cardioprotective effect by converting angiotensin II to angiotensin (1–7)^(10–12)^ and through the direct effect of angiotensin-(1–7) binding to the Mas receptor and in such a manner exerts vasodilatory effects contrary to angiotensin II^(13)^.

ACE2 exists as a membrane-bound form and a soluble form (sACE) and both forms possess catalytic activity and bind to SARS-CoV and SARS-CoV-2^(14,15)^. The binding of these viruses to sACE2 in circulation will prevent binding to ACE2 in the membrane and inhibit infection^(14–16)^. Since sACE2 lacks the membrane anchor, it cannot promote viral entry into the cell and replication. Thus, sACE2 acts as a competitive interceptor of SARS-CoV and SARS-CoV-2, and increasing the amount of sACE2 has been suggested as a promising potential therapy^(17)^.

The ACE2-receptor is required for cell entry for three coronaviruses; SARS-CoV-2^(1)^, SARS-CoV^(18)^ and HCov-NL63^(19)^. Development of drugs or identification of natural substances that bind to ACE2 and disrupt the interaction with the spike protein on coronaviruses could potentially be of great value to prevent the development of severe disease following coronavirus infection, and studies so far indicate that chronic inhibition of ACE2 is safe^(20)^. Promising results were reported for the synthetic ACE2 inhibitor N-(2-aminoethyl)-1 aziridine-ethanamine, which prevented SARS-CoV spike protein-mediated cell fusion^(21)^, but this compound has not been tested clinically. Several *in vitro* studies have shown that ACE activity can be inhibited by peptides from fish and other marine organisms^(22,23)^, and based on the similarities in the amino acid sequence between ACE and ACE2, we presumed that also the ACE2 activity may be inhibited by basic amino acids or small peptides^(24,25)^. Dietary proteins are hydrolysed to smaller peptides before absorption in the gastrointestinal tract, and a large number of bioactive peptides have been identified in common foods. Several ACE-inhibitory peptides have been found in fish and seafood^(26)^; however, peptides with ACE2-inhibiting activities have not yet been identified in foods of marine origin.

A recent study using data from 2884 front-line healthcare workers from six Western countries presents evidence that diet may be an important factor for the course of COVID-19, since following plant-based or pescatarian diets was associated with lower odds of moderate-to-severe COVID-19^(27)^. A protective effect of fish may be due to the long-chain *n*-3 polyunsaturated fatty acids found in marine animals, since supplementation with marine fatty acids reduced the need for mechanical ventilation and reduced hospitalisation time at intensive care units for patients with acute respiratory distress syndrome^(28)^. Other nutrients in fish such as vitamin D have been suggested to prevent severe COVID-19, although no prophylactic or therapeutic role of vitamin D in COVID-19 has been confirmed^(29)^, and a recent Norwegian study found no protective effect of supplementation with cod liver oil (containing n-3 PUFA EPA and DHA, and vitamins D3, A and E) in the winter on SARS-CoV-2 infection or serious COVID-19^(30)^. People following plant-based or pescatarian diets may be in general more conscious of their health and have a healthier lifestyle and a stronger immune system, with a lower prevalence of nutrient deficiencies for long-chain *n*-3 PUFA, Zn, Se and vitamins C, D and E which is beneficial for the immune system^(31)^. As of yet, the possible protective effect of foods of marine origin on the development of severe COVID-19 has not been investigated.

Several approaches have been suggested to address possible therapeutic prevention of COVID-19, including spike-based vaccines, inhibition of ACE2 or TMPRSS2, and increasing the sACE2 concentration^(32)^. In the present study, we aimed to test the effect of consuming Atlantic cod muscle on the sACE2 concentration in serum from rats. Cod was chosen since this is a commonly consumed fish. We also explored the *in vitro* ACE2 inhibitory potential of various fractions from four marine fish species; haddock, saithe, Atlantic cod and golden redfish. Our hypothesis was that fish intake would increase the sACE2 concentration in rats, and that fish protein powders were able to inhibit ACE2 activity *in vitro*.

## Methods

### Ethical statement

The National Animal Research Authority (Norway) approved the protocol for the rat study in accordance with the Animal Welfare Act and the Regulation of animal experiments (approval no 2014/6979). All applicable international, national and institutional guidelines for the care and use of animals were followed. The fish used in this study were captured through regular fisheries and were frozen onboard.

### Rat study

Twelve male Zucker fa/fa rats (HsdHlr:ZUCKER-Leprfa, from Harlan Laboratories) were randomly assigned to two experimental groups, i.e., the Cod group or the Control group, of six rats, each with comparable mean body weight. The rats were housed in pairs in Makrolon IV cages in a room maintained at a 12-h light–dark cycle (light from 7 a.m. to 19.00) with a constant temperature of 20–23°C and relative humidity of 65 ± 15 %. The rats were acclimatised under these conditions before the start of the experiment, and the intervention started when the rats were 8–9 weeks old. Rats had free access to feed, drinking water, wood chewing sticks and plastic housing.

The rats were fed modified experimental diets in accordance with the American Institute of Nutrition’s recommendation for growing laboratory rodents (AIN-93G)^(33)^, added 1·6 g methionine/kg diet and 1 wt% growth and maintenance supplement containing vitamin B_12_ and vitamin K1 as recommended by Reeves^(34)^. The diets differed only in their protein sources; the Cod diet contained 5 wt% proteins from cod muscle (Atlantic cod (*Gadus morhua*) provided by Lerøy Seafood Group) and 15 wt% proteins from casein. Skin-free cod fillets were baked (180°C for 20 min), minced, and thereafter lyophilised and ground. The Control diet contained 20 wt% proteins from casein. Diets were frozen immediately after preparation. Casein was purchased from Sigma-Aldrich, and the other feed ingredients were purchased from Dyets Inc. Feed was given as a powder formula and was contained in ceramic bowls. Newly thawed feed was provided every day except Sundays (rat were given double doses on Saturdays).

The rats were killed after 4 weeks of intervention while under anaesthesia with isoflurane (Isoba vet, Intervet, Schering-Plough Animal Health) mixed with oxygen and nitrous oxide, after an overnight fast with free access to drinking water. Blood was drawn from the heart and was collected in Vacuette Z Serum Clot Activator Tubes (Greiner Bio-one) for isolation of serum. Serum aliquots were snap-frozen in liquid nitrogen and stored at –80°C until analysis. Assessors responsible for general daily animal care, euthanasia and analyses of samples were blinded to diet groups, and rats were handled in random order.

Serum concentrations of sACE2, angiotensin II, TNF-*α* and IL-6 were quantified using the following assays: Rat ACE2/ACE-2 ELISA Kit (Sandwich ELISA, LS-F33787 from LifeSpan BioSciences, Inc.), Rat Angiotensin II ELISA Kit (Competitive EIA, LS-F3953 from LifeSpan BioSciences), Rat TNF-alpha ELISA Kit (ELR-TNFa from RayBiotech Inc) and Interleukin-6 (rat) ELISA kit (EIA-4845 from DRG Instruments GmbH). All samples were analysed simultaneously in the same plate from each of the assays. The mean within-plate coefficient of variation (CV) were 4·6 % for ACE2, 2·2 % for angiotensin II, 4·5 % for TNF-*α*, and 4·3 % for IL-6. The plates were read at 450 nm on a SpectraMax Plus384 Microplate Reader (Molecular Devices).

### Analysis of ACE2 inhibiting activity *in vitro*

Haddock (*Melanogrammus aeglefinus*), saithe (*Pollachius virens*), Atlantic cod (*Gadus morhua*) and golden redfish (*Sebastes norvegicus*) were captured in the Barents Sea in October 2020 and were frozen at −30°C on-board the industry trawler Granit (Halstensen Granit). The fish were landed and then partially thawed overnight at ambient temperature. Next, the fish were headed, gutted and filleted, and the individual fractions (muscle, backbone, skin, head, stomach (rinsed with cold tap water), stomach content, intestine (rinsed with cold tap water) and swim bladder) were lyophilised. Muscles, backbones and heads were minced before drying. The dried samples were milled and kept at −20°C until analyses.

The dried fish powders were added Trizma buffer (50 mM, pH 8·0, all ingredients from Sigma) and were hydrolysed using trypsin from bovine pancreas (T1426 from Sigma) at 45°C for 4 h^(35)^. The hydrolysates were heated to 90°C for 20 min to inactivate trypsin and any endogenous ACE2. Next, samples were cooled in ice water for 30 min and frozen at –20°C until analyses. Protein content in the hydrolysates was quantified on the Cobas c111 system (Roche Diagnostics GmbH) using the TP2 kit from Roche.

ACE2 inhibition was measured using the ACE2 Inhibitor Screening Assay Kit (Item No. 502 100 from Cayman Chemical) as described in the user manual. In brief, 75 μl assay buffer, 10 μl human recombinant ACE2 enzyme, 5 μl protein hydrolysate or ACE2 inhibitor MLN-4760 (as positive control) and 10 μl of substrate (7-methoxycoumarin-4-yl)acetyl-Ala-Pro-Lys(2,4-dinitrophenyl) were added to half-volume 96-well solid black plates. ACE2 cleaves the substrate and produces free (7-methoxycoumarin-4-yl)acetyl which is fluorescent. Samples were run in duplicates. Plates were incubated for 30 min at room temperature and read with an excitation wavelength of 320 nm and an emission wavelength of 405 nm on a SpectraMax Gemini EM Fluorescence Microplate Reader (Molecular Devices). Inter-assay CV was 0·5 %, and the mean within-plate CV was 0·4 %.

### Outcome measurements

The primary outcome of this project was to investigate the *in vivo* effect of fish intake on sACE2 in rats. The secondary outcome was to examine the *in vitro* ACE2-inhibiting activity of fish protein powders.

### Statistical analyses

Since this is the first study to investigate the effects of cod fillet intake on sACE2 concentration in rats, data on effect size were not available for sample size calculation or minimally detectable effect sizes for the present study.

Statistical analyses were conducted using SPSS Statistics version 25 (SPSS, Inc., IBM Company). The serum concentrations of sACE2, angiotensin II and TNF-*α* were log-transformed and the experimental groups were compared using unpaired Student’s t test. ANOVA was used to compare the fish fractions, followed by the Tukey HSD post hoc test to determine significant differences between fractions when appropriate. The cut-off value for statistical significance was set at a probability of 0·05.

## Results

### sACE2, angiotensin II, TNF-*α* and IL-6 in rat serum

**Fig. 1.**
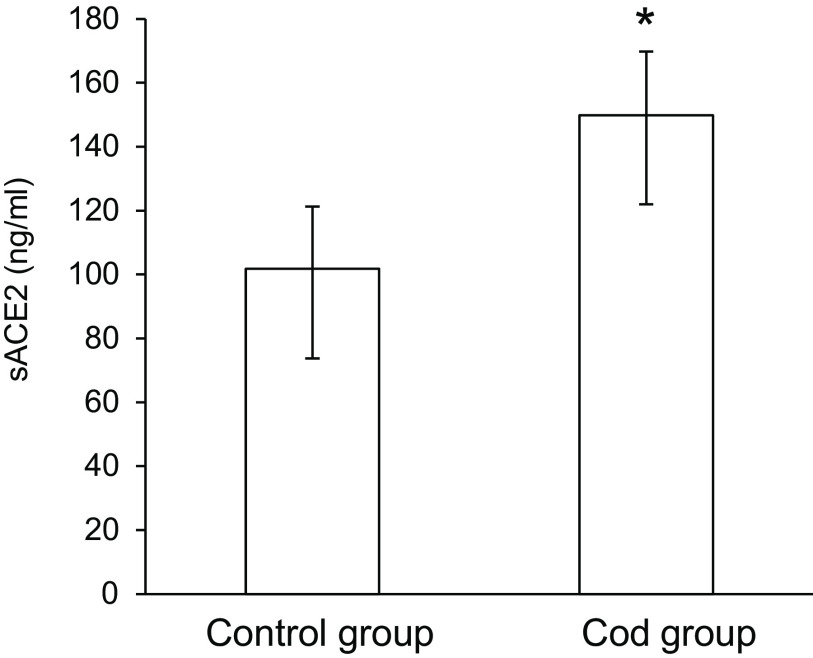
Serum concentrations of sACE2 in rats fed a Cod diet or a Control diet. The bars represent the geometric means with quartiles for six rats in each group. *Geometric mean was significantly different from that of the Control group (*P* < 0·05, evaluated by unpaired Student’s *t* test).

The body weight was similar between the Control group and the Cod group at baseline (357 ± 14 g and 354 ± 10 g, respectively, P 0·64) and after 4 weeks of intervention (570 ± 21 g and 578 ± 14 g, respectively, P 0·49). The geometric mean for the serum concentration of sACE2 was significantly higher in rats fed cod muscle when compared with the Control group (47 % higher, P 0·034, Fig. 1), with no differences between the groups for angiotensin II (P 0·55, data not presented). The serum concentration of TNF-*α* was similar between the Control group and the Cod group (P 0·46, data not presented), whereas the serum IL-6 concentration was below the lowest concentration in the standard curve (31·3 pg/ml) for all rats and were not analysed statistically.

### ACE2 inhibition

**Fig. 2.**
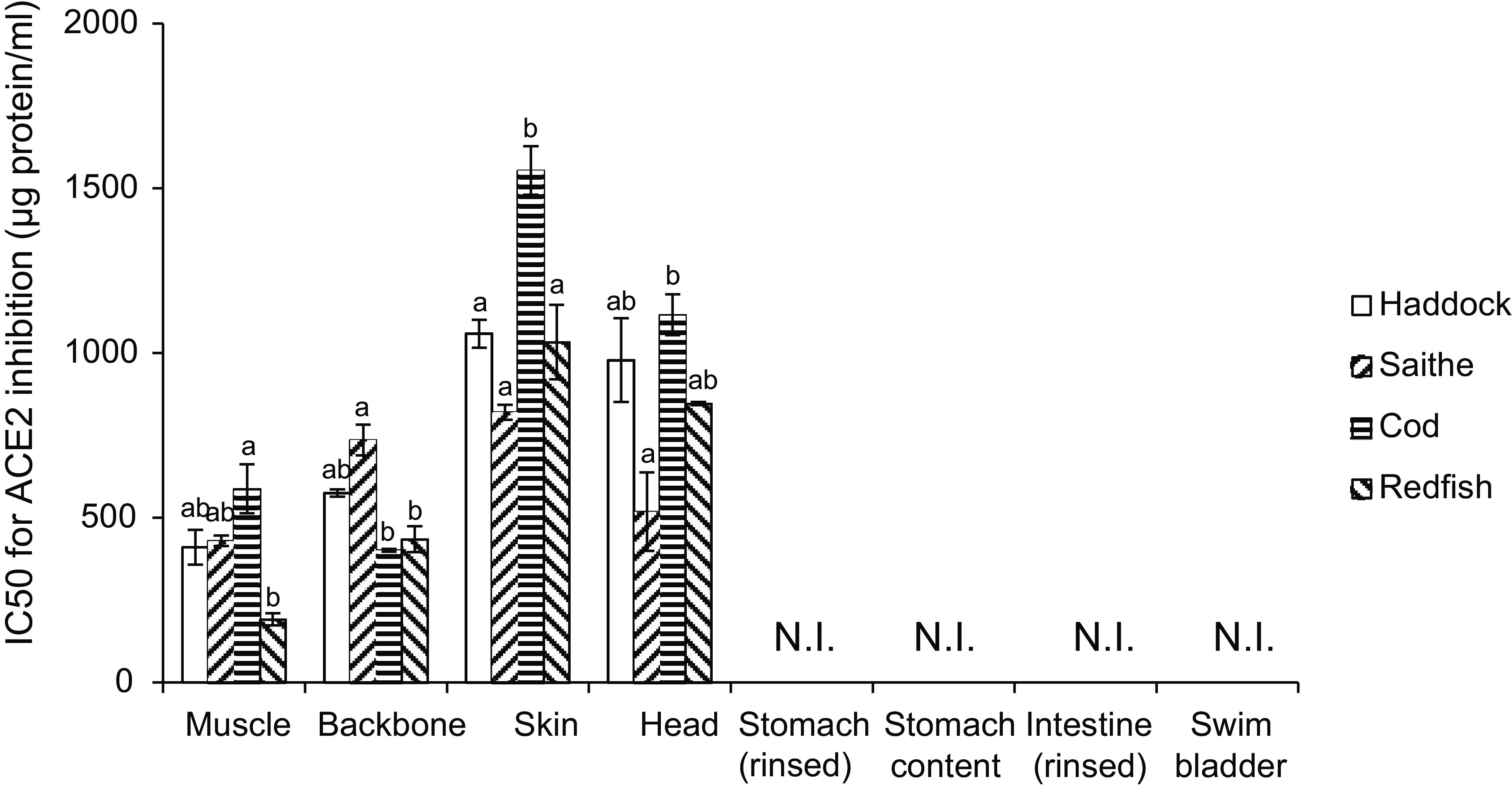
IC50 values for ACE2 inhibition by fish protein hydrolysates prepared with trypsin. The bars represent the amount of protein in µg/ml needed to inhibit 50 % of the ACE2 activity. Data are presented as the mean with their standard error of mean shown by vertical bars for two measurements. The hydrolysates were compared fraction-wise using one-way ANOVA with Tukey HSD post hoc test, and different letters indicate significant differences between protein hydrolysates; *P* < 0·05 was considered significant. N.I.: no inhibition detected.

Trypsin-hydrolysed muscle, backbone, head and skin fractions from haddock, saithe, Atlantic cod and golden redfish inhibited ACE2 activity, whereas no inhibition of ACE2 was seen for stomach, stomach content, intestine and swim bladder hydrolysates from any of the tested fish species (Fig. 2). We compared the four fish species for individual fractions and found no difference between haddock and saithe for muscle, backbone, skin and head. For muscle, golden redfish was a more potent ACE2-inhibitor compared with Atlantic cod, but was not different from haddock and saithe. The backbone fraction from Atlantic cod and golden redfish were more potent ACE2 inhibitors compared with that of saithe, but were not significantly different from haddock backbone. Atlantic cod skin was the least efficient ACE2 inhibitor compared with skin fractions from the other fish species. For the head fraction, saithe was more efficient in inhibiting ACE2 when compared with Atlantic cod, but none of these were different from haddock and golden redfish.

The ACE2-inhibiting activity of the muscle, backbone, head and skin fractions from the four tested fish species were compared using ANOVA (P 7·4 × 10^–9^) with Tukey HSD post hoc test. The homogeneous subset output showed that hydrolysates of muscles and backbones from all four fish species in addition to saithe head were more potent ACE2-inhibitors compared with the rest of the samples. The Atlantic cod skin fraction had the lowest capacity for ACE2 inhibition when compared with all other fractions (the p-values for these comparisons are not displayed in Fig. 2).

## Discussion

This study is the first to present evidence for the potential of fish muscle to increase the serum concentration of sACE2 when consumed by rats, thereby suggesting that dietary intake of fish may inhibit cell entry and replication of SARS-CoV-2 and thus reduce COVID-19 infection. The prospect of increasing sACE2 through changes in diet has never before been explored. We also show that hydrolysates of muscle, backbone, skin and head fractions from haddock, saithe, Atlantic cod and golden redfish possess ACE2-inhibitory properties *in vitro*.

sACE2 protects from lung injury and blocks cell entry of SARS-CoV-2^(15,16,36)^ and has a cardioprotective effect by preventing angiotensin II-induced hypertension and cardiac fibrosis^(37)^. Increasing the amount of sACE2 has been suggested as a promising potential therapy for coronavirus infection^(17)^, and clinical phase I and phase II trials show that sACE2 administration is well tolerated in healthy subjects^(38)^ and in patients with acute respiratory distress syndrome^(39)^. The membrane-bound ACE2 is cleaved by TMPRSS2 upon binding of SARS-CoV-2 to enable entry into the cell or by sheddases to release sACE2. The main sheddase for ACE2 is a disintegrin and metallopeptidase domain 17 (ADAM17)^(40)^, which also releases membrane-bound pro-inflammatory cytokines including TNF-*α*, IL-6^(41)^ and interferon-*γ* ^(42)^. ADAM17 is activated by increased binding of angiotensin II to the angiotensin II type 1 receptor^(43)^, and the serum concentration of soluble ADAM17 was shown to be higher in severely ill COVID-19 patients^(44)^. Importantly, the sACE2 activity was markedly elevated and was associated with a higher IL-6 concentration in patients with severe COVID-19^(45)^. Increased shedding of ACE2 due to activation of the renin–angiotensin system following upregulation of ADAM-17 also drives the pathogenesis in diseases such as heart failure and coronary artery disease^(46)^, and elevated plasma sACE concentration is associated with a greater risk of major CVD events^(47)^. We believe that the elevated sACE2 concentration observed in rats fed cod muscle is not associated with activation of ADAM17, since both angiotensin II and TNF-*α* serum concentrations were similar between the experimental groups, and the IL-6 concentration was below quantification level for all rats. This is also supported by previous observations of no signs of increased inflammation in obese Zucker fa/fa rats after dietary treatment with Atlantic cod^(48)^ or blue whiting (*Micromesistius poutassou*)^(49)^, both members of the Gadidae family. Further, adult study participants with overweight/obesity consuming five weekly dinners with cod fillet actually had lower serum concentrations of neopterin (stimulated by interferon-*γ* during immune activation)^(50)^, with no changes in serum concentrations of TNF-*α*, IL-8 and monocyte chemoattractant protein 1^(51)^.

The current rat study was not designed to provide a definitive description of the mechanism(s) behind the higher sACE2 concentration in rats consuming a diet containing cod muscle, but we speculate that intake of cod may stimulate the gene expression of ACE2 through inhibition of ACE activity. This suggestion is based on studies showing that plasma ACE2 activity was increased in COVID-19 patients treated with a pharmacological ACE inhibitor^(52)^ and that ACE inhibitors increased cardiac ACE2 gene transcription in Lewis rats^(53)^. Peptides with ACE inhibitory activity have been discovered in several fish species, including cod^(26)^, and we have previously shown that hydrolysed cod muscle efficiently inhibits ACE activity *in vitro* ^(54)^.

The possibility that peptides in common foods may prevent COVID-19 infection is inspiring and should be thought-provoking. Bioactive peptides taken orally are absorbed through the intestine and will, if intact, enter the blood for transport and may induce systemic effects or trigger local effects in the gastrointestinal tract. Although di- and tripeptides are quickly absorbed in the intestine, they may not always reach their target organs or cells, as a consequence of the action of proteases in the gastrointestinal tract resulting in them being hydrolysed to inactive peptides or free amino acids. Also, larger peptides may be hydrolysed into bioactive peptides in the intestine. For peptides to have an effect on ACE2 activity, this will require that the bioactive peptides that are liberated during enzymatic digestion of proteins can be absorbed intact in the intestine and transported by the blood to their sites of action. Small peptides taken orally have been retrieved in their intact form in plasma in humans^(55)^ and may thus impact biochemical systems and physiological processes. The search for specific bioactive peptides in the blood that originate from the diet is challenging due to the abundance of peptides of endogenous origin and from exogenous sources. A better approach *in vivo* may be to investigate the direct effects of individual peptides. Studies have shown that oral administration of several (but by no means all) single peptides with ACE-inhibiting activity *in vitro* have an acute lowering effect on the blood pressure in spontaneously hypertensive rats^(56–63)^. Oral administration of tripeptides for 1 week^(60)^ or 18 d^(64)^ also reduced the blood pressure in spontaneously hypertensive rats. These studies, and other studies using single peptides or mixtures of peptides, strongly indicate that some bioactive peptides resist degradation and thus are bioavailable and able to exhibit physiological activity when they reach their target cells. It is possible that the occurrence of circulating ACE2 inhibitors observed in human blood by Lew *et al.* ^(24)^ may have originated from the diet after consumption of food such as fish containing bioactive motifs or peptides with ACE2-inhibiting properties, and it was suggested that these might be basic amino acids or small peptides. This strengthens the concept that peptides with ACE2-inhibiting effects from consumed fish may be absorbed and bind to ACE2, thus inhibiting the activity of ACE2, in the human body.

It was recently shown that following plant-based or pescatarian diets protected against moderate-to-severe COVID-19^(27)^. Fish contains a plethora of important nutrients, such as minerals and vitamins that are essential for a well-functioning immune system^(31)^, in addition to essential fatty acids and high-quality proteins. Here, we investigated the *in vitro* ACE2 inhibiting potential of protein hydrolysates prepared from different parts of fish, including the muscle which represents the most popular part of the fish in Western cuisine. We observed that the ACE2 inhibitory capacity was evident for muscles from all four fish species tested, which makes these interesting candidates for future *in vivo* research. While fish’ muscles are readily prepared for dinner, the backbone, skin and head fractions (which also showed ACE2-inhibiting capacity) may be more challenging to include in an everyday diet. We therefore suggest that the use of dried powders prepared from the rest raw materials should be further investigated as supplements and enrichments to common foods.

The present study has several limitations. In the rat study, only one fraction from one single fish species, i.e. muscle from cod, was investigated, and a generalisation regarding an effect of fish intake on serum sACE2 concentration cannot be based on this study alone. For the *in vitro* study, we mimicked protein digestion by using only trypsin, thus the ACE2-inhibiting peptides that were liberated from the fish proteins may be different from those released during digestion in the human gastrointestinal tract. Although sACE2 concentration was increased after cod muscle consumption in rats, we have no knowledge on whether the activity of sACE2 may be inhibited by fish intake, which is a possibility since fish protein powders inhibited ACE2 activity *in vitro*. The concentration and the activity of sACE2 *in vivo* are not necessarily associated, which may be due to, amongst other things, the presence of endogenous inhibitors, thus it is important to gain more knowledge about the effect of fish intake on the activity of ACE2. Our research group will continue with testing of various fish species as part of a regular diet in humans to investigate if an increase in sACE2 concentration and activity can be obtained in normal weight or overweight humans with no or low-grade systemic inflammation, and to investigate if sACE2 activity *in vivo* may be affected by fish consumption.

### Conclusion

In the present study, we show for the first time that fish intake increases the serum concentration of sACE2 in rats and that fish proteins have ACE2-inhibiting potential *in vitro*. The increase in sACE2 was probably not due to increased shedding as a consequence of ADAM17 activation, since neither angiotensin II, TNF-*α* nor IL-6 was increased in rats fed fish muscle, but may be a consequence of inhibition of ACE activity by the fish proteins. These findings indicate that dietary fish intake may protect against COVID-19 infection. Further research should investigate a potential association between fish intake and the severity of COVID-19 in humans, and whether fish should be recommended as part of a healthy diet to prevent COVID-19 infection.

## References

[1] Hoffmann M , Kleine-Weber H , Schroeder S , et al. (2020) SARS-CoV-2 cell entry depends on ACE2 and TMPRSS2 and is blocked by a clinically proven protease inhibitor. Cell 181, 271–280.e278. 32142651 10.1016/j.cell.2020.02.052 PMC7102627

[2] Matsuyama S , Nagata N , Shirato K , et al. (2010) Efficient activation of the severe acute respiratory syndrome coronavirus spike protein by the transmembrane protease TMPRSS2. J Virol 84, 12658–12664. 20926566 10.1128/JVI.01542-10 PMC3004351

[3] Bertram S , Heurich A , Lavender H , et al. (2012) Influenza and SARS-coronavirus activating proteases TMPRSS2 and HAT are expressed at multiple sites in human respiratory and gastrointestinal tracts. PLoS One 7, e35876. 22558251 10.1371/journal.pone.0035876 PMC3340400

[4] Popkin BM , Du S , Green WD , et al. (2020) Individuals with obesity and COVID-19: a global perspective on the epidemiology and biological relationships. Obes Rev 21, e13128. 32845580 10.1111/obr.13128 PMC7461480

[5] Page-Wilson G , Arakawa R , Nemeth S , et al. (2021) Obesity is independently associated with septic shock, renal complications, and mortality in a multiracial patient cohort hospitalized with COVID-19. PLoS One 16, e0255811. 34383798 10.1371/journal.pone.0255811 PMC8360607

[6] Sarver DC & Wong GW (2021) Obesity alters Ace2 and Tmprss2 expression in lung, trachea, and esophagus in a sex-dependent manner: implications for COVID-19. Biochem Biophys Res Commun 538, 92–96. 33168188 10.1016/j.bbrc.2020.10.066 PMC7605802

[7] Verdecchia P , Cavallini C , Spanevello A , et al. (2020) The pivotal link between ACE2 deficiency and SARS-CoV-2 infection. Eur J Intern Med 76, 14–20. 32336612 10.1016/j.ejim.2020.04.037 PMC7167588

[8] Donoghue M , Hsieh F , Baronas E , et al. (2000) A novel angiotensin-converting enzyme-related carboxypeptidase (ACE2) converts angiotensin I to angiotensin 1–9. Circ Res 87, E1–E9. 10969042 10.1161/01.res.87.5.e1

[9] Tipnis SR , Hooper NM , Hyde R , et al. (2000) A human homolog of angiotensin-converting enzyme. Cloning and functional expression as a captopril-insensitive carboxypeptidase. J Biol Chem 275, 33238–33243. 10924499 10.1074/jbc.M002615200

[10] Kassiri Z , Zhong J , Guo D , et al. (2009) Loss of angiotensin-converting enzyme 2 accelerates maladaptive left ventricular remodeling in response to myocardial infarction. Circ Heart Fail 2, 446–455. 19808375 10.1161/CIRCHEARTFAILURE.108.840124

[11] Yamamoto K , Ohishi M , Katsuya T , et al. (2006) Deletion of angiotensin-converting enzyme 2 accelerates pressure overload-induced cardiac dysfunction by increasing local angiotensin II. Hypertens 47, 718–726. 10.1161/01.HYP.0000205833.89478.5b 16505206

[12] Crackower MA , Sarao R , Oudit GY , et al. (2002) Angiotensin-converting enzyme 2 is an essential regulator of heart function. Nature 417, 822–828. 12075344 10.1038/nature00786

[13] Santos RA , Simoes e Silva AC , Maric C , et al. (2003) Angiotensin-(1–7) is an endogenous ligand for the G protein-coupled receptor Mas. Proc Natl Acad Sci USA 100, 8258–8263. 12829792 10.1073/pnas.1432869100 PMC166216

[14] Jia HP , Look DC , Tan P , et al. (2009) Ectodomain shedding of angiotensin converting enzyme 2 in human airway epithelia. Am J Physiol Lung Cell Mol Physiol 297, L84–L96. 19411314 10.1152/ajplung.00071.2009 PMC2711803

[15] Monteil V , Kwon H , Prado P , et al. (2020) Inhibition of SARS-CoV-2 infections in engineered human tissues using clinical-grade soluble human ACE2. Cell 181, 905–913.e907. 32333836 10.1016/j.cell.2020.04.004 PMC7181998

[16] Leow MKS (2020) Clarifying the controversial risk-benefit profile of soluble ACE2 in COVID-19. Crit Care 24, 396 32631373 10.1186/s13054-020-03097-w PMC7338146

[17] Batlle D , Wysocki J & Satchell K (2020) Soluble angiotensin-converting enzyme 2: a potential approach for coronavirus infection therapy? Clin Sci 134, 543–545. 10.1042/CS20200163 32167153

[18] Li W , Moore MJ , Vasilieva N , et al. (2003) Angiotensin-converting enzyme 2 is a functional receptor for the SARS coronavirus. Nature 426, 450–454. 14647384 10.1038/nature02145 PMC7095016

[19] Hofmann H , Pyrc K , van der Hoek L , et al. (2005) Human coronavirus NL63 employs the severe acute respiratory syndrome coronavirus receptor for cellular entry. Proc Natl Acad Sci USA 102, 7988–7993. 15897467 10.1073/pnas.0409465102 PMC1142358

[20] Montanari M , Canonico B , Nordi E , et al. (2021) Which ones, when and why should renin-angiotensin system inhibitors work against COVID-19? Adv Biol Regul 81, 100820. 34419773 10.1016/j.jbior.2021.100820 PMC8359569

[21] Huentelman MJ , Zubcevic J , Hernandez Prada JA , et al. (2004) Structure-based discovery of a novel angiotensin-converting enzyme 2 inhibitor. Hypertension 44, 903–906. 15492138 10.1161/01.HYP.0000146120.29648.36

[22] Abachi S , Bazinet L & Beaulieu L (2019) Antihypertensive and angiotensin-I-Converting Enzyme (ACE)-inhibitory peptides from fish as potential cardioprotective compounds. Mar Drugs 17, 613. 31671730 10.3390/md17110613 PMC6891548

[23] Pujiastuti DY , Ghoyatul Amin MN , Alamsjah MA , et al. (2019) Marine organisms as potential sources of bioactive peptides that inhibit the activity of angiotensin I-converting enzyme: a review. Molecules 24, 2541. 31336853 10.3390/molecules24142541 PMC6680877

[24] Lew RA , Warner FJ , Hanchapola I , et al. (2008) Angiotensin-converting enzyme 2 catalytic activity in human plasma is masked by an endogenous inhibitor. Exp Physiol 93, 685–693. 18223027 10.1113/expphysiol.2007.040352 PMC7197901

[25] Clayton D , Hanchapola I , Thomas WG , et al. (2015) Structural determinants for binding to angiotensin converting enzyme 2 (ACE2) and angiotensin receptors 1 and 2. Front Pharmacol 6, 5. 25688208 10.3389/fphar.2015.00005 PMC4311625

[26] Ngo DH , Vo TS , Ngo DN , et al. (2012) Biological activities and potential health benefits of bioactive peptides derived from marine organisms. Int J Biol Macromol 51, 378–383. 22683669 10.1016/j.ijbiomac.2012.06.001

[27] Kim H , Rebholz CM , Hegde S , et al. (2021) Plant-based diets, pescatarian diets and COVID-19 severity: a population-based case-control study in six countries. BMJ Nutr Prev Health 4, 257–266. 10.1136/bmjnph-2021-000272 PMC8219480 34308134

[28] Dushianthan A , Cusack R , Burgess VA , et al. (2019) Immunonutrition for acute respiratory distress syndrome (ARDS) in adults. Cochrane Database Syst Rev 1, CD012041. 10.1002/14651858.CD012041.pub2 PMC6353063 30677127

[29] Brito DTM , Ribeiro LHC , Daltro C , et al. (2021) The possible benefits of vitamin D in COVID-19. Nutrition 91–92, 111356 10.1016/j.nut.2021.111356 PMC8149468 34352586

[30] Brunvoll SH , Nygaard AB , Ellingjord-Dale M , et al. (2022) Prevention of covid-19 and other acute respiratory infections with cod liver oil supplementation, a low dose vitamin D supplement: quadruple blinded, randomised placebo controlled trial. BMJ 378, e071245 36215222 10.1136/bmj-2022-071245 PMC9449357

[31] Shakoor H , Feehan J , Al Dhaheri AS , et al. (2021) Immune-boosting role of vitamins D, C, E, zinc, selenium and *n*-3 fatty acids: could they help against COVID-19? Maturitas 143, 1–9. 33308613 10.1016/j.maturitas.2020.08.003 PMC7415215

[32] Zhang H , Penninger JM , Li Y , et al. (2020) Angiotensin-converting enzyme 2 (ACE2) as a SARS-CoV-2 receptor: molecular mechanisms and potential therapeutic target. Intens Care Med 46, 586–590. 10.1007/s00134-020-05985-9 PMC7079879 32125455

[33] Reeves PG , Nielsen FH & Fahey GC Jr (1993) AIN-93 purified diets for laboratory rodents: final report of the American Institute of Nutrition writing committee on the reformulation of the AIN-76A rodent diet. J Nutr 123, 1939–1951. 8229312 10.1093/jn/123.11.1939

[34] Reeves PG (1996) AIN-93 purified diets for the study of trace element metabolism in rodents. In Trace Elements in Laboratory Rodents pp. 3–37 [ RR Watson , editor]. Boca Raton, FL: CRC Press Inc.

[35] Shalaby SM , Zakora M & Otte J (2006) Performance of two commonly used angiotensin-converting enzyme inhibition assays using FA-PGG and HHL as substrates. J Dairy Res 73, 178–186. 16476177 10.1017/S0022029905001639

[36] Heurich A , Hofmann-Winkler H , Gierer S , et al. (2014) TMPRSS2 and ADAM17 cleave ACE2 differentially and only proteolysis by TMPRSS2 augments entry driven by the severe acute respiratory syndrome coronavirus spike protein. J Virol 88, 1293–1307. 24227843 10.1128/JVI.02202-13 PMC3911672

[37] Huentelman MJ , Grobe JL , Vazquez J , et al. (2005) Protection from angiotensin II-induced cardiac hypertrophy and fibrosis by systemic lentiviral delivery of ACE2 in rats. Exp Physiol 90, 783–790. 16049057 10.1113/expphysiol.2005.031096

[38] Haschke M , Schuster M , Poglitsch M , et al. (2013) Pharmacokinetics and pharmacodynamics of recombinant human angiotensin-converting enzyme 2 in healthy human subjects. Clin Pharmacokinet 52, 783–792. 23681967 10.1007/s40262-013-0072-7

[39] Khan A , Benthin C , Zeno B , et al. (2017) A pilot clinical trial of recombinant human angiotensin-converting enzyme 2 in acute respiratory distress syndrome. Crit Care 21, 234. 28877748 10.1186/s13054-017-1823-x PMC5588692

[40] Lambert DW , Yarski M , Warner FJ , et al. (2005) Tumor necrosis factor-α convertase (ADAM17) mediates regulated ectodomain shedding of the severe-acute respiratory syndrome-coronavirus (SARS-CoV) receptor, angiotensin-converting enzyme-2 (ACE2). J Biol Chem 280, 30113–30119. 15983030 10.1074/jbc.M505111200 PMC8062222

[41] Scheller J , Chalaris A , Garbers C , et al. (2011) ADAM17: a molecular switch to control inflammation and tissue regeneration. Trends Immunol 32, 380–387. 21752713 10.1016/j.it.2011.05.005

[42] Dreymueller D , Pruessmeyer J , Groth E , et al. (2012) The role of ADAM-mediated shedding in vascular biology. Eur J Cell Biol 91, 472–485. 22138087 10.1016/j.ejcb.2011.09.003

[43] Patel VB , Clarke N , Wang Z , et al. (2014) Angiotensin II induced proteolytic cleavage of myocardial ACE2 is mediated by TACE/ADAM-17: a positive feedback mechanism in the RAS. J Mol Cell Cardiol 66, 167–176. 24332999 10.1016/j.yjmcc.2013.11.017

[44] Palacios Y , Ruiz A , Ramon-Luing LA , et al. (2021) Severe COVID-19 Patients Show an Increase in Soluble TNFR1 and ADAM17, with a Relationship to Mortality. Int J Mol Sci 22, 8423. 34445140 10.3390/ijms22168423 PMC8395100

[45] Reindl-Schwaighofer R , Hodlmoser S , Eskandary F , et al. (2021) ACE2 elevation in severe COVID-19. Am J Respir Crit Care Med 203, 1191–1196. 33600742 10.1164/rccm.202101-0142LE PMC8314901

[46] Gheblawi M , Wang K , Viveiros A , et al. (2020) Angiotensin-converting enzyme 2: SARS-CoV-2 receptor and regulator of the renin-angiotensin system: celebrating the 20th anniversary of the discovery of ACE2. Circ Res 126, 1456–1474. 32264791 10.1161/CIRCRESAHA.120.317015 PMC7188049

[47] Narula S , Yusuf S , Chong M , et al. (2020) Plasma ACE2 and risk of death or cardiometabolic diseases: a case-cohort analysis. Lancet 396, 968–976. 33010842 10.1016/S0140-6736(20)31964-4 PMC7529405

[48] Drotningsvik A , Mjos SA , Hogoy I , et al. (2015) A low dietary intake of cod protein is sufficient to increase growth, improve serum and tissue fatty acid compositions, and lower serum postprandial glucose and fasting non-esterified fatty acid concentrations in obese Zucker fa/fa rats. Eur J Nutr 54, 1151–1160. 25380663 10.1007/s00394-014-0793-x

[49] Drotningsvik A , Oterhals A , Mjos SA , et al. (2021) Effects of intact and hydrolysed blue whiting proteins on blood pressure and markers of kidney function in obese Zucker fa/fa rats. Eur J Nutr 60, 529–544. 32409916 10.1007/s00394-020-02262-9 PMC7867508

[50] Helland A , Bratlie M , Hagen IV , et al. (2021) Effect of high intake of cod or salmon on serum total neopterin concentration: a randomised clinical trial. Eur J Nutr 60, 3237–3248. 33576844 10.1007/s00394-021-02497-0 PMC8354862

[51] Helland A , Bratlie M , Hagen IV , et al. (2017) High intake of fatty fish, but not of lean fish, improved postprandial glucose regulation and increased the *n*-3 PUFA content in the leucocyte membrane in healthy overweight adults: a randomised trial. Br J Nutr 117, 1368–1378. 28606215 10.1017/S0007114517001234

[52] Kintscher U , Slagman A , Domenig O , et al. (2020) Plasma angiotensin peptide profiling and ACE (Angiotensin-Converting Enzyme)-2 activity in COVID-19 patients treated with pharmacological blockers of the renin-angiotensin system. Hypertension 76, e34–e36. 32851897 10.1161/HYPERTENSIONAHA.120.15841 PMC7480797

[53] Ferrario CM , Jessup J , Chappell MC , et al. (2005) Effect of angiotensin-converting enzyme inhibition and angiotensin II receptor blockers on cardiac angiotensin-converting enzyme 2. Circulation 111, 2605–2610. 15897343 10.1161/CIRCULATIONAHA.104.510461

[54] Vildmyren I , Drotningsvik A , Oterhals A , et al. (2018) Cod residual protein prevented blood pressure increase in zucker fa/fa rats, possibly by inhibiting activities of angiotensin-converting enzyme and renin. Nutrients 10, 1820–1833. 30469459 10.3390/nu10121820 PMC6315726

[55] Shen W & Matsui T (2017) Current knowledge of intestinal absorption of bioactive peptides. Food Funct 8, 4306–4314. 29139513 10.1039/c7fo01185g

[56] Saito Y , Wanezaki K , Kawato A , et al. (1994) Antihypertensive effects of peptide in sake and its by-products on spontaneously hypertensive rats. Biosci Biotechnol Biochem 58, 812–816. 7764971 10.1271/bbb.58.812

[57] Fujita H & Yoshikawa M (1999) LKPNM: a prodrug-type ACE-inhibitory peptide derived from fish protein. Immunopharmacol 44, 123–127. 10.1016/s0162-3109(99)00118-6 10604535

[58] Nakashima Y , Arihara K , Sasaki A , et al. (2002) Antihypertensive activities of peptides derived from porcine skeletal muscle myosin in spontaneously hypertensive rats. J Food Sci 67, 434–437.

[59] Sato M , Hosokawa T , Yamaguchi T , et al. (2002) Angiotensin I-converting enzyme inhibitory peptides derived from wakame (Undaria pinnatifida) and their antihypertensive effect in spontaneously hypertensive rats. J Agric Food Chem 50, 6245–6252. 12358510 10.1021/jf020482t

[60] Suetsuna K , Maekawa K & Chen JR (2004) Antihypertensive effects of Undaria pinnatifida (wakame) peptide on blood pressure in spontaneously hypertensive rats. J Nutr Biochem 15, 267–272. 15135150 10.1016/j.jnutbio.2003.11.004

[61] Nii Y , Fukuta K , Yoshimoto R , et al. (2008) Determination of antihypertensive peptides from an izumi shrimp hydrolysate. Biosci Biotechnol Biochem 72, 861–864. 18323650 10.1271/bbb.70565

[62] Vercruysse L , Van Camp J , Morel N , et al. (2010) Ala-Val-Phe and Val-Phe: ACE inhibitory peptides derived from insect protein with antihypertensive activity in spontaneously hypertensive rats. Peptides 31, 482–488. 19524628 10.1016/j.peptides.2009.05.029

[63] Escudero E , Toldra F , Sentandreu MA , et al. (2012) Antihypertensive activity of peptides identified in the *i*n vitro gastrointestinal digest of pork meat. Meat Sci 91, 382–384. 22405912 10.1016/j.meatsci.2012.02.007

[64] Majumder K , Chakrabarti S , Morton JS , et al. (2015) Egg-derived ACE-inhibitory peptides IQW and LKP reduce blood pressure in spontaneously hypertensive rats. J Funct Foods 13, 50–60.

